# Investigating the immune mechanism of natural products in the treatment of lung cancer

**DOI:** 10.3389/fphar.2024.1289957

**Published:** 2024-02-14

**Authors:** Lian Yang, Yukun Chen, Kaile Liu, Yuanyuan Chen, Yu Zhang, Zhanxia Zhang, Hegen Li

**Affiliations:** ^1^ Department of Oncology, Longhua Hospital, Shanghai University of Traditional Chinese Medicine, Shanghai, China; ^2^ Department of Oncology, Dongfang Hospital, Beijing University of Chinese Medicine, Beijing, China; ^3^ Cancer Institute, Longhua Hospital, Shanghai University of Traditional Chinese Medicine, Shanghai, China

**Keywords:** natural products, lung cancer, antitumor immunity, adaptive immunity, innate immunity

## Abstract

With the deepening of people’s understanding of lung cancer, the research of lung cancer immunotherapy has gradually become the focus of attention. As we all know, the treatment of many diseases relies on the rich sources, complex and varied compositions and wide range of unique biological properties of natural products. Studies have shown that natural products can exert anticancer effects by inducing tumor cell death, inhibiting tumor cell proliferation, and enhancing tumor cell autophagy. More notably, natural products can adjust and strengthen the body’s immune response, which includes enhancing the function of NK cells and promoting the differentiation and proliferation of T lymphocytes. In addition, these natural products may enhance their anticancer effects by affecting inhibitory factors in the immune system, hormone levels, enzymes involved in biotransformation, and modulating other factors in the tumor microenvironment. The importance of natural products in lung cancer immunotherapy should not be underestimated. However, the specific links and correlations between natural products and lung cancer immunity are not clear enough, and further studies are urgently needed to clarify the relationship between the two. In this paper, we will focus on the correlation between natural products and lung cancer immune responses, with a view to providing new research perspectives for immunotherapy of lung cancer.

## Graphical Abstract

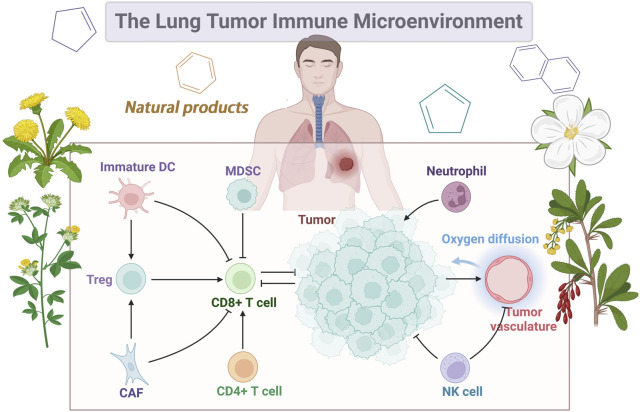

## 1 Introduction

One of the deadliest malignant tumors worldwide is lung cancer, which is commonly divided into two primary forms: small-cell lung cancer (SCLC) and non-small cell lung cancer (NSCLC) (Zheng, 2016). Surgery and radiotherapy are the most frequently used clinical approaches for lung cancer treatment. However, there remains a significant chance of lingering cancer cells following the surgical procedure and a propensity for the reappearance or spread of the disease (Hoy et al., 2019). Chemotherapy and radiation therapy, frequently accompanied by adverse reactions like hematopoietic suppression, harm to the liver and kidneys, gastrointestinal dysfunction, and in rare instances, can exacerbate the illness and jeopardize the anticipated result (Duma et al., 2019; Chaft et al., 2022). Lung cancer treatment has changed significantly, and in the past, clinical practice often relied on uniform guidelines and standardized protocols. However, modern lung cancer treatment focuses more on multidisciplinary teamwork and the integrated use of surgery, radiotherapy, chemotherapy, targeted therapy, and immunotherapy to achieve the best possible outcome, with immunotherapy playing an important role that is increasingly recognized and valued (Hirsch et al., 2017).

The main drugs of tumor immunotherapy include tumor vaccines, Chimeric Antigen Receptor T-cell immunotherapy (CAR-T), and immune checkpoint inhibitors (ICIs) (Sterner and Sterner, 2021). Studies suggest that the immune system or immune surveillance of a person plays a crucial part in the progression and growth of lung cancer (Lahiri et al., 2023). Both the innate and adaptive immune systems can initiate immune reactions against lung cancer. Consequently, immunotherapy for lung cancer has transitioned from solely serving as adjuvant treatment to becoming a vital strategy in its management (Vollmers and Brändlein, 2009). ICIs offer a superior and less harmful approach to immunotherapy, whether used alone or in conjunction with radiotherapy or various targeted drugs (Zhou et al., 2021). The progress made in this area has greatly enhanced the advancement of immunotherapy for lung cancer.

Nevertheless, lung cancer often exhibits resistance to immunologic agents, thereby restricting the extensive and prolonged application of immunotherapy in its treatment (Hermanowicz et al., 2020). Currently, the majority of immunologic agents utilized in clinical practice fall under the classification of biologics (Ohaegbulam et al., 2015). In contrast to biologics, small molecule tumor immunotherapeutic agents can enter cell membranes and target intracellular sites. Numerous small molecule metabolites have been documented to hinder the interaction between programmed death receptor 1 (PD-1) and programmed cell death 1 ligand 1(PD-L1) molecules (Offringa et al., 2022; Yamaguchi et al., 2022). The development time and cost of chemically synthesized small molecules are increasing, however. Natural products are pharmacologically active metabolites derived from various plants, animals, or microorganisms (Wang et al., 2022a). Natural products offer a number of advantages over biologics, such as a wider range of biological activities, lower side effects and a greater diversity of potent ingredients (Ma et al., 2021). An extensive array of pharmaceutical companies are increasingly focusing on extracting medicinal plants to discover new and powerful natural drugs and their derivatives, and to develop novel medications (He et al., 2022). Organic substances possess the unique ability to not just inhibit the proliferation and dissemination of cancer and other malignant cells but also enhance the body’s immune reactions against tumors (Miller and Hanna, 2021; Huo et al., 2022; Islam et al., 2022). The distinctive benefits it offers could potentially enhance ongoing tumor immunotherapy efforts and create new opportunities for research and development in this field. Exploring and utilizing natural products for immunotherapy of lung cancer is of great significance. In this article, we will provide an overview of the immunomodulatory functions of natural substances in the treatment of lung cancer. This includes their impact on innate immunity, acquired immunity, and immune checkpoints. Enhancing the literary proof for the utilization of natural substances in the clinical immunotherapy of lung cancer is an extensively researched field (Demaria et al., 2019; Sivori et al., 2021).

## 2 Innate immunity

The body’s main defense against foreign pathogens and cancer cells is the innate immune system, which plays a vital role in protecting overall health. Macrophages, a type of innate immune cell, carry out important functions by participating in processes like phagocytosis and cytotoxic activities. Dendritic cells (DCs) are responsible for processing and presenting antigens to initiate acquired immunity, while NK cells can directly recognize and eliminate tumor cells. On the other hand, myeloid-derived suppressor cells (MDSCs) are a diverse group of bone marrow-derived cells that suppress the activity of other immune cells. Over the past few years, extensive research efforts have been focused on understanding the function of MDSCs in the suppressive tumor microenvironment and revealing the molecular mechanisms that govern them (Sui et al., 2022). Therefore, bolstering the inherent immune system emerges as a successful strategy in the fight against cancer.

### 2.1 Macrophage regulation

Macrophages consist of a distinct group of innate immune cells and are present in the majority of body tissues. The growth of tumors is directly related to the density of macrophages, making it a crucial focus for cancer treatment (Yunna et al., 2020). Tumor-associated macrophages (TAM) playing an active role in regulating blood vessel formation, altering the extracellular matrix, promoting cancer cell growth, spreading, and suppressing the immune system (Chen et al., 2019). Macrophages can differentiate into two separate phenotypes, referred to as M1 and M2. Classically activated M1 macrophages possess the ability to generate cytotoxic agents like nitric oxide, reactive oxygen species, and inflammatory mediators. These metabolites function to combat tumor cells, either directly or indirectly, by triggering the activation of immune cells like T cells and NK cells. They also contribute to anti-tumor immune responses, ultimately playing a vital part in restricting tumor advancement (Xia et al., 2020). M2 macrophages, also known as alternatively activated macrophages, can generate substances like arginine, anti-inflammatory agents, growth factors, and angiogenic factors. These substances aid in the survival, growth, and spread of cancer cells, contribute to immunosuppressive reactions, and facilitate tumor formation and progression (Zhu et al., 2015). Recent research has demonstrated that converting TAM from an M2 state to an M1 state can effectively enhance targeted anti-cancer immunity and suppress tumor metastasis. Moreover, recent inquiries have discovered the presence of PD-1 on TAM, which hinders their ability to engulf particles and their immune functions against tumors. Therefore, the activation of M1 macrophages or the suppression of M2 macrophages has become a vital approach in the fight against cancer (Gao et al., 2022).

Studies have shown that Rhodiola algida (Crassulaceae; Roots) release of granulocyte-macrophage colony-stimulating factors, IL-2, and IL-4, resulting in increased mRNA levels and the promotion of lymphocyte proliferation (Loo et al., 2010). Additionally, Rhodiola rosea extract prompts RAW264.7 macrophages to produce NO, thereby slowing down tumor progression (Jia et al., 2023). It can be inferred that Rhodiola rosea has a positive regulatory effect on the immune system. Increasing the release of these immune factors and the proliferation of lymphocytes helps to improve the body’s defense against pathogens and abnormal cells. In addition, rhodiola rosea stimulates the production of NO by macrophages, which helps to slow down the progression of tumors has an important regulatory role in the immune system and may play a role in inhibiting the growth of tumor cells. The bioactive substance Astragaloside IV (Fabaceae; Astragalus root), which is present in Astragalus, effectively inhibited the M2 polarization in macrophages that was induced by IL-13 and IL-4. This resulted in a reduction in CD206 expression and a decrease in the expression of genes related to M2 polarization. Furthermore, Astragaloside IV impeded the invasion, migration, and angiogenesis induced by M2-conditioned media in A549 and H1299 cells (Saeedifar et al., 2021). Moreover, Astragaloside IV treatment led to a reduction in the number of M2 macrophages present in tumor tissues (Xu et al., 2018). Astragaloside IV achieves macrophage regulation by interfering with the regulation at the level of key molecules, showing its possible antitumor effects in the regulation of the tumor microenvironment, which provides insight into its mechanism of action and a theoretical basis for future drug design. The potential of Ginsenosides (Araliaceae; Rhizome) as agents to combat lung cancer cells is considerable. Studies have uncovered that Ginsenosides can regulate the differentiation of TAM and their interactions in the tumor microenvironment. Indirect immunomodulation leads to anti-tumor activity as a consequence of these impacts. Research has confirmed that Ginsenosides can decrease the levels of CD206, which is a marker for M2 macrophages, and diminish the expression of VEGF, MMP2, and MMP9 in lung cancer cells when they are co-cultured with Ginsenosides (Im, 2020). Thus, Ginsenosides may inhibit the development and spread of lung cancer by regulating these key molecules. Ginsenosides exert their antitumor activity through multifaceted effects (modulation of TAM, reduction of CD206, reduction of VEGF, MMP, etc. expression). This suggests that for drug or compound research, integrating multiple pathways and targets may be more effective in achieving therapeutic effects. Certain natural products achieve their anti-tumor effects by inhibiting angiogenesis, and their mechanisms of action against tumors are intricately connected to macrophages. Tripterygium wilfordii (Ranunculaceae; Seed and bark) main metabolite, celastrol, inhibits the expression of CD206, arginase 1, and CD204. It also decreases the release of anti-inflammatory cytokines, resulting in a decrease in the number of tumor-associated M2-polarized macrophages and hindering tumor angiogenesis (Wang et al., 2019). Celastrol affected the differentiation status of M2-type macrophages by interfering with the expression of CD206, CD204 and others. This reminds us that specific cellular phenotypes and molecular markers may be important therapeutic targets in cancer therapy and are expected to be modulated to achieve anti-cancer effects.

### 2.2 Enhancing DCs

DCs play a crucial role in the immune system by presenting antigens and are essential for both innate and adaptive immune responses. The effectiveness of anti-cancer immune responses heavily relies on the operational condition of DCs. Enhancing the function of DCs in individuals with cancer to induce activation of T-cells is a powerful approach in the field of anti-tumor immunotherapy. The reason for this is that mature DCs secrete IL-12, which subsequently affects T cells and encourages the development of Th1 cells (Type 1 helper T cells) (Gardner et al., 2020). Researchers have observed that over 80% of exosomes derived from lung cancer biopsy tissues contain EGFR, and DCs can capture these exosomes. Nonetheless, this capture event results in the differentiation of DCs into tolerogenic DCs. Afterward, these immune cells with suppressive properties can then prompt the transformation of initial CD4^+^ T cells into regulatory T cells (Tregs) specific to tumor antigens, ultimately hindering the activity of CD8^+^ T cells that target the tumor (Stevens et al., 2020). To summarize, these results indicate that exosomes originating from tumors allow tumor cells to escape detection by the immune system. The presence of this occurrence could potentially be utilized as a target for the advancement of immunotherapeutic approaches in the treatment of cancer (Zhang and Yu, 2019).

The polysaccharide from Astragalus (Fabaceae; Roots) stimulated the transformation of splenic DCs into DCs with high levels of CD11c and low levels of CD45RB, and also converted Th2 cells into Th1 cells, thereby boosting the immune function of T lymphocytes (Liu et al., 2011). This reminds us that we need to pay attention to the functions and changes of DCs when studying immune regulation mechanisms. In addition, the maturation of DCs was enhanced by both purified Glycyrrhetinic acid (Fabaceae; Rhizome) and Red Ginseng extracts (Araliaceae; Rhizome), thereby improving their ability to combat tumors. The previous enhanced the production of IL-12 and IL-10 while decreasing the levels of TNF-α. IFN-γ secretion by splenic T lymphocytes stimulated the release of TNF-α and IL-1β levels in mice with tumors (Kim et al., 2011; Cho et al., 2019), which was caused by plant polysaccharides. One such polysaccharide is Achyranthes bidentata polysaccharide (Amaranthaceae; Roots), which is derived from Achyranthes bidentata and is renowned for its surprising immunomodulatory effects. The metabolite was discovered to cause phenotypic maturation in DCs and enhance IL-12 production by boosting the levels of CD86, CD40, and MHC II expression. As a result, it activates DCs in mice (Zou et al., 2011). Cordyceps sinensis (Ophiocordycipitaceae; Cordyceps sinensis fruiting body), which is used in clinical settings to treat lung cancer and different lung ailments, acts as a stimulant and promoter of immature DCs. By enhancing the production of co-stimulatory molecules and pro-inflammatory cytokines in DCs, it boosts the proliferation of allogeneic T cells driven by DCs, while simultaneously reducing the endocytic capability of DCs (Li et al., 2009). The generalization of these effects should be questioned, taking into account the variability of individual responses or potential side effects.

### 2.3 Activating NK cells

NK cells, a crucial part of the innate immune system, are well-known for their rapid identification and elimination of infected cells, which makes them an essential factor in the body’s protection against viral infections and cancer. Natural killer cells can initiate immune responses against cancerous cells without requiring specific antigens. The potential of NK cell-based immunotherapy in fighting tumors has been emphasized by both experimental and clinical research (Tang J. et al., 2022a). Cancer progression and patient mortality are significantly influenced by the spread of cancer cells to distant sites. The ability of cancer cells to spread to other areas involves natural mechanisms that enable them to invade the surrounding environment, enter the bloodstream, and form colonies in distant places. In this scenario, NK cells have a crucial function in controlling the spread and multiplication of cancer cells, aiding in the goal of stopping tumor development (Chan and Ewald, 2022).

Several research studies have emphasized the capacity of organic substances to regulate the development and spread of tumors by stimulating natural killer cells. The polysaccharide from Ganoderma lucidum (Ganodermataceae; Fruiting body) has demonstrated the ability to enhance the quantity and cytotoxicity of natural killer cells, along with the activity of cytotoxic T lymphocytes in mice. The tumor size is decreased and the death of tumor cells is encouraged by this complex carbohydrate, possibly by activating caspase-3 and caspase-9 (Chien et al., 2004). This result suggests that Ganoderma lucidum polysaccharides may induce tumor cell death by activating caspase-3 and caspase-9. However, whether this explanation has been confirmed by detailed molecular biology and cell biology experiments and whether there are other possible explanations needs to be explored in more depth. Astragalus (Fabaceae; Roots) and Angelica (Apiaceae; Rhizome), when combined, have been found in recent studies to hinder the growth of lung cancer, promote the differentiation of NK and Tc cells, and reinstate the equilibrium of immune cell ratios and cytokine profiles within the tumor microenvironment (Wu et al., 2019). The description mentions possible mechanisms for the combination of Astragalus and Angelica, as well as the regulation of immune cell ratios and the balance of cytokine profiles in the tumor microenvironment. Although experimentally confirmed, the existence of other possible explanations needs to be explored in more depth and the rationality of the ratio of the two drugs needs to be experimentally analyzed.

### 2.4 Suppression of MDSCs

Pluripotent hematopoietic stem cells in physiological conditions differentiate and transition from immature myeloid cells (IMCs) to mature myeloid cells, exhibiting a wide range of functions (Li et al., 2021). The myeloid cells can be classified into three different groups: macrophages, DCs and granulocytes. They have vital functions in maintaining the normal operations of the immune system, acting as protectors against harmful infections, eliminating dead cells, and aiding in tissue restructuring through immune responses (Yuo, 2001).

A diverse population of bone marrow mesenchymal stem cells consists of myeloid progenitor cells and IMCs. Under normal conditions, IMCs naturally differentiate into mature bone marrow-derived cells like macrophages, DCs or neutrophils. However, when there is inflammation, infection, or neoplastic disorders, the usual differentiation of IMCs is disrupted, resulting in the formation of MDSCs (Charbord, 2010; Naji et al., 2019). MDSCs mainly consist of immature monocytes/macrophages, DCs, granulocytes, and other myeloid cells in early differentiation stages, commonly known as natural suppressor cells. MDSCs play a vital role and exhibit powerful immunosuppressive abilities within the tumor microenvironment (Veglia et al., 2018). These cells, which originate in the bone marrow, then migrate to peripheral lymphoid organs and tumor sites in the host, where they play a role in creating an immunosuppressive environment. MDSCs enhance the advancement of tumors by aiding in the survival of tumor cells, stimulating the formation of new blood vessels, and facilitating the invasion and spread of cancer to healthy tissues (Kumar et al., 2016).

Research has uncovered the capacity of different organic substances to impact mesenchymal stem cells found in bone marrow. *In vitro* experiments revealed that Asparagus polysaccharide (Asparagaceae; Tender stems and tender shoots) effectively hindered the growth of MDSCs and triggered their programmed cell death via the TLR4 pathway. In MDSCs, Asparagus polysaccharide increased Bax and caspase-9 expression levels while inhibiting Bcl-2 expression, showing significant anti-MDSC properties and alleviating its inhibition of the immune system against tumors (Zhang et al., 2018). The results were obtained under double-blind experimental conditions and reached a sufficient sample size to be statistically significant. In another investigation, Lin and colleagues. It was documented that Ginseng (Araliaceae; Rhizome) and Atractylodes macrocephala (Asteraceae; Rhizome) decreased the infiltration of MDSCs and inhibited the epithelial-mesenchymal transition caused by transforming growth factor-β1, in addition to activating Wnt5a (Guo et al., 2015). The combined results emphasize the possibility of using natural substances to regulate the growth of tumors by focusing on MDSCs, providing hopeful opportunities in the management of cancer.

## 3 Acquired immune

The adaptive immune system is a precise immune response that targets and eliminates particular antigens, such as harmful bacteria and cancerous cells. Studies suggest that the active metabolites present in organic substances can have a suppressive impact on tumor growth by affecting the specialization of T-cell groups and regulating the release of cytokines (Vesely et al., 2011).

### 3.1 Regulation of T lymphocyte upregulation

T cells, which are a crucial metabolite of the human immune system, have a significant impact on identifying specific symptoms and determining the body’s resistance capabilities. These T cells can be divided into two main groups: CD4^+^ T cells and CD8^+^ T cells, which can be distinguished by the presence of CD4 and CD8 antigenic proteins (Taniuchi, 2018). CD4^+^ T cells, also known as Helper T cells, and CD8^+^ T cells, also referred to as cytotoxic T cells, both have crucial functions in the context of anti-tumor immunity (Borst et al., 2018). Cytotoxic T lymphocytes (CTL), a key functional subset of CD8^+^ T cells, can directly eradicate cancerous cells. On the other hand, the most abundant subset of T cells is CD4^+^ T cells, which primarily function to coordinate and regulate immune responses. Different subsets can be formed by CD4^+^ T cells, such as Th1, Th2, Th17, Treg, and others. Among these, Th1 cells play a major role in cellular immune responses by stimulating macrophages and NK cells, thereby enhancing cytotoxicity. On the other hand, Th2 lymphocytes primarily secrete cytokines like IL-4, IL-5, IL-6, IL-10, and IL-13, actively participating in humoral immune responses through the activation of B cells and the promotion of antibody synthesis (Raskov et al., 2021; Speiser et al., 2023). There is a noticeable difference in lung cancer patients compared to healthy individuals. In particular, there is a reduction in the levels of Th1-type cytokines, specifically IFN-γ and IL-2, while Th2-type cytokines such as IL-4, IL-6, and IL-13 demonstrate an elevation. The difference highlights the significance of Th1/Th2 imbalance in the development of cancer (Weidemüller et al., 2021; Alagbe et al., 2022).

The proliferation of Th1 cells can be promoted by certain natural substances, which may result in antitumor effects. It has been shown that Curcumin (Zingiberaceae; Rhizome) converts Foxp3^+^ regulatory T cells to T helper type 1 cells in lung cancer patients. Curcumin treatment in lung cancer patients resulted in a decrease in the percentage of peripheral blood regulatory T cells while increasing the percentage of peripheral blood T helper type 1 cells (Zou et al., 2018). The study lacks specific information on the dose, treatment method, and placebo details. The short 2-week treatment period raises uncertainty about long-term effects. The absence of specific statistical results and sample sizes for lung cancer patients and healthy subjects hinders the assessment of result reliability. In a comparative investigation, when Tetramethylpyrazine (Lamiaceae; Rhizome) was introduced into peripheral blood mononuclear cell cultures derived from both lung cancer patients and healthy individuals, noteworthy outcomes emerged. The existence of Tetramethylpyrazine resulted in increased productions of interferon-γ and IL-2 and decreased quantities of Th2-type cytokines. Additionally, it boosted the cytotoxic capability of peripheral blood mononuclear cells obtained from individuals with lung cancer (Wei et al., 2002). There was some key information missing from the study, such as the inclusion of a healthy control group and details of the experimental design, which could have an uncertain impact on the reliability of the results. In addition, the article mentions the expression levels of T-bet and GATA3 mRNA but does not delve into their specific relationship with Th1 and Th2 drift. The production of interferon-γ and IL-2 was enhanced by Ginsenosides (Araliaceae; Rhizome), which stimulated DCs to facilitate the transformation of initial T cells into Th1-type cells, consequently leading to an increase in interferon-γ production. In cytotoxicity assays, Ginsenosides induced higher interferon-γ than mature DCs. Therefore, combining Ginsenosides and DCs for tumor treatment may induce stronger Th1 immunity (You et al., 2022). Although the study mentioned that the metabolites of M1 and M4 may be the actual active substances of ginsenosides *in vivo*, it did not delve into the specific mechanism of action of these metabolites. Secondly, the study mainly focused on *in vitro* experimental conditions, while more in-depth studies on the effects on the immune response *in vivo* are needed. Furthermore, conducted research revealed that the administration of Ginsenoside Rg3 (Araliaceae; Rhizome) led to a significant rise in interferon-γ and IL-2 concentrations in mice with tumors, in comparison to the control group (Liu et al., 2019). This study used mice as a model to investigate the immune effects of Rg3, but it should be noted that the mouse immune system differs from that of humans. Although Rg3 was experimentally shown to be protective against CTX-induced immunosuppression, more studies are needed to evaluate its safety and efficacy for its prospective clinical application, especially clinical trials with large patient populations. This indicates that Astragalus effectively addressed the imbalance of Th1 and Th2 cytokines. Significantly, the activation of STAT3 has been linked to the proliferation and spread of tumors. The growth of lung cancer was significantly reduced by Resveratrol [Polygonaceae; Grape Skin], a metabolite rich in polyphenols, which achieved this by inhibiting M2-like polarization among TAM and decreasing p-STAT3 expression within tumor tissues (Sun et al., 2017).

Treg cells, or regulatory T cells, are a special subset of T lymphocytes in the immune system that play a key role in regulating immune system activity and maintaining immune balance. Treg cells, as important mediators of the immune system, are mainly responsible for inhibiting the activity of other T lymphocytes and immune cells to ensure an appropriate response of the immune system and have immunosuppressive properties (Tanaka and Sakaguchi, 2019). The relationship between Treg cells and tumorigenesis, progression, and prognosis is significant. Targeting Treg cells *in vivo* with therapeutic methods can enhance the body’s anti-tumor immunity (Whiteside, 2018). Natural products have the potential to enhance anti-tumor immunity, inhibit tumor cell growth, and reduce metastasis by reducing the number and function of Treg cells, as well as the immunosuppressive cytokines they produce (Li et al., 2012). It also has an immunomodulatory impact by suppressing the expression of transforming growth factor-β through activation of the TLR4 signaling pathway, thereby decreasing Treg cell levels. The article states that CXCR4 is the only target gene for AS-IV, but this is only a prediction based on BATMAN. It is important to note that the predictions are preliminary clues, not definitive conclusions. Detailed parameters and accuracy assessment of BATMAN predictions are not provided. The need for more large sample clinical trials to validate the findings is mentioned, which is reasonable. However, specific clinical trial plans and expected results were not detailed, which is necessary information. Rehmannia glutinosa polysaccharides [Scrophulariaceae; Roots] decrease the presence of CD3^+^ and CD8^+^ cells, stimulate the immune function of lymphocytes, regulate cytokines like TNF-α, IL-17, IFN-γ, IL-4, and IL-10, and enhance monocyte phagocytosis (Zeng et al., 2019). Different research discovered that Echinacea (Asteraceae; Flower and Leaf) decreased the quantity of CD4^+^, CD25^+^, and Foxp3^+^ Treg cells, diminished their inhibitory capability, and improved the function of antigen-presenting cells. However, it did not have a direct impact on the proliferation of T cells (Kim et al., 2014). Due to technical difficulties, the study focused on investigating the inhibitory function of CD4^+^ and CD25^+^ cells and did not directly isolate live FoxP3^+^ cells. This experimental design is to some extent justified, but the reasons for the choice and its potential impact on experimental conclusions need to be clarified. The limited studies on the mechanisms of attenuation of Tregs number and function provide a plausible explanation that elevated IL-6 levels in EP-treated mice may be associated with a reduced frequency of Tregs, but this is a hypothesis that needs to be supported by more experimental evidence.

## 4 Immune checkpoints

Lately, the realm of immune intervention techniques focused on immune checkpoint molecules like PD-1 and cytotoxic t-lymphocyte associated antigen 4 (CTLA4) has offered fresh hope for treating associated disorders, especially cancer. Preliminary inquiries have revealed the presence of specific immunosuppressive metabolites expressed on effector T cells, which function to inhibit excessive activation of T cells and the resulting emergence of autoimmune conditions. As a result, widespread attention has been attracted to the role of these molecules in regulating immune balance (Tang et al., 2022b). Additional examination has revealed more information regarding the increased presence of these checkpoint molecules in circumstances like cancerous growths, persistent viral infections, and bacterial infections within immune cells. These molecules assume pivotal roles in the pathophysiology of these conditions. Significantly, the major immune checkpoint molecules include CTLA4, PD-1, TIM3, and LAG3. PD-1, an essential co-repressor molecule identified in the last 10 years, has become a central controller of T-cell activity (Lei et al., 2020). Furthermore, PD-1 is found in the exteriors of different immune cells, such as B cells, monocytes/macrophages, NK cells, and DCs, where it functions in the negative regulation of their activities (Yi et al., 2022). Tumor cells frequently demonstrate increased PD-L1 molecule expression in particular tumor microenvironments. By binding to and blocking the activation of PD-1 molecules on infiltrating T cells, these PD-L1 molecules allow tumors to escape attacks from the immune system. Disruption of this binding by monoclonal antibodies that target PD-1 or PD-L1 can restore the anti-tumor function of T cells. The field of cancer immunotherapy has been greatly advanced by these findings (Han et al., 2020). There are reports that Platycodon grandiflorum (PG) (Campanulaceae; Roots) can reduce the expression of PD-1 on the surface of CD8^+^ T cells, thereby exerting an anti-tumor effect in NSCLC. The potential mechanism involves an increase in the infiltration and cytotoxic activity of CD8^+^ T cells by PG, which is associated with the reduction of PD-1 in CD8^+^ T cells. Additionally, PG regulates the expression of PD-1 on the surface of CD8^+^ T cells by reducing the secretion of VEGF-A in tumor cells, which is regulated by the levels of phosphorylated signal transducer and activator of transcription 3 (P-STAT3) (Yang et al., 2022). Molecular docking was performed in the thesis using the Glide software, but no details of the specific parameters and XP scoring model were provided, which has a significant impact on the accuracy of the docking results, and more details need to be provided. Molecular docking calculations were performed three times to calculate the mean and standard deviation, which is good practice, but it is not stated whether the initial conditions were the same for each calculation, and it is vital to ensure consistency. More explanations and experimental evidence are needed regarding the detailed mechanisms by which PG regulates PD-1 expression and the effects on P-STAT3 levels and VEGF-A secretion in tumor cells. Andrographolide (AD) is a diterpenoid lactone compound extracted from Andrographis paniculate (Apiaceae; Leaves and young stems). Studies have shown that treating H1975 and H1299 cells with AD significantly inhibits the expression of PD-L1 protein and mRNA and can also reduce PD-L1 levels induced by IFN-γ. The potential mechanism might be that the reactive oxygen species (ROS) produced by AD inhibit the JAK2-STAT3 signaling pathway in NSCLC, leading to the accumulation of P62, which in turn regulates the expression of PD-L1 (Wang et al., 2022b). This paper lacks a detailed discussion and experimental validation of the molecular mechanisms. Although molecular dynamics simulations suggest that AD binds to STAT3 with high affinity, there is insufficient information about the simulation parameters, stability, and reliability. Detailed information is essential to assess the credibility of the simulation results. The article notes the improved efficacy of AD at higher concentrations but lacks discussion of potential toxicity at different doses, which is critical for clinical application. Although AD enhances anti-PD-1 antibody immunotherapy, there is insufficient detail on the specific immunomodulatory mechanisms, effects on CD8^+^ T cells function, and relationship to PD-L1 downregulation. Recent studies have revealed that Evodiamine exhibits exceptional efficacy in inhibiting PD-L1 expression induced by interferon-gamma (IFN-γ), particularly notable in H1975 and H1650 cell lines. Further research indicates that Evodiamine (Rutaceae; Gains) significantly reduces the expression of MUC1-C mRNA and protein in NSCLC cells. Concurrently, Evodiamine downregulates PD-L1 expression, decreases T-cell apoptosis, suppresses MUC1-C expression, and enhances the effector function of CD8^+^ T cells. These findings underscore the immense potential of Evodiamine as a prospective therapeutic agent for NSCLC (Jiang et al., 2020). The paper mentions that Evodiamine attenuated IFN-γ-induced PD-L1 expression, however, the exact immunomodulatory mechanisms and signaling pathways need to be investigated in greater detail. The combination treatment of evodiamine with anti-PD-1 antibody enhanced tumor growth control and mouse survival in the Lewis lung cancer model. However, the detailed mechanisms and potential adverse effects of this combination therapy were not discussed in depth in the paper. Ginsenoside Rg3 not only hinders the proliferation of lung cancer cells but also reduces their resistance to cisplatin. This is achieved by decreasing the expression of PD-L1 induced by chemoresistance and enhancing the cytotoxicity of T-cells towards cancer cells. Furthermore, Ginsenoside Rg3 exerts its effects on NF-κB p65 and Akt, both of which play a role in the excessive expression of PD-L1, thereby suppressing their function. The characteristic of Ginsenoside Rg3 renders it a possible therapeutic choice for chemoresistant refractory NSCLC (Jiang et al., 2017). The article points out that Rg3 influences immune escape by regulating PD-L1. For the molecular mechanism of PD-L1 regulation, the article only mentions that the expression of NF-κB and Akt is affected, but does not delve into their specific roles in PD-L1 regulation, but does not explain in sufficient detail the intracellular signaling pathways and molecular mechanisms in the experiment. In addition, the article used A549/DDP cells as study subjects but did not fully explain the reason for choosing this cell model. Sometimes, the choice of a cell line may affect the applicability and generalization of the study. Berberine (Berberidaceae; Roots or rhizomes), a metabolite found in different plants and belonging to the isoquinoline alkaloid family, has been employed for the management of cancer, bacterial infections, diabetes, cardiovascular diseases, and inflammatory disorders. In addition to its recognized applications, berberine has been discovered to function as a suppressor of PD-L1. Berberine increases the responsiveness of tumor cells to co-cultured T cells in lung cancer therapy by decreasing the levels of PD-L1 in cancer cells. Additionally, it enhances the immune response of tumor-infiltrating T cells and reduces the activation of immunosuppressive MDSCs and Tregs, resulting in antitumor effects in mice with Lewis lung tumor xenografts (Liu et al., 2020). The results showed that the immunomodulatory function of BBR was mainly manifested at a dose of 4 mg/kg. This emphasizes the importance of dosage, as too high a dose may lead to adverse effects such as suppressed mitochondrial energy metabolism and weight loss. BBR promotes anti-tumor T-cell immunity by decreasing the expression of PD-L1 in MDSCs and Tregs, which is in line with previous studies and has important implications for clinical immunotherapy.

## 5 Conclusion

In recent years, with the vigorous advancement of drug research and immunotherapy, the study of natural products has gradually become a focal point. Natural products originate from plants, microorganisms, or animals, and their natural origin endows them with higher potential safety. Compared to synthetic drugs, they typically reduce the risk of adverse effects because of their higher affinity to biological entities. Natural products contain various bioactive ingredients such as alkaloids, polysaccharides, glycosides, and flavonoids, demonstrating extensive biological activity. These components can have multifaceted and complex effects on the immune system, covering both innate and adaptive immunity.

Some synthetic drugs may face the challenge of drug resistance, while natural products, due to their complex bioactive components, generally have a lower risk of developing resistance. Natural products often consist of multiple components that may influence multiple cellular pathways, resulting in comprehensive therapeutic effects. This aids in addressing the complexity of lung cancer. The diversity of natural products allows them to impact lung cancer immunity through multiple pathways, providing a more comprehensive inhibition of cancer cell growth and dissemination.

However, natural products also have some drawbacks in the treatment of lung cancer immunity compared to synthetic drugs, introducing certain differences. The complex chemical composition and diverse molecular structures of natural products often make it challenging to achieve consistent therapeutic effects among different individuals. In contrast, the structure of synthetic drugs is more easily controlled, facilitating the attainment of consistent efficacy. Additionally, the bioavailability of natural products may be limited due to potential influences from *in vivo* metabolism and absorption. In comparison, synthetic drugs can enhance their bioavailability by adjusting chemical structures, thereby increasing therapeutic effectiveness.

Natural products may affect multiple cells or receptors, whereas synthetic drugs can achieve more precise therapeutic effects by designing molecules with greater specificity. This helps reduce unnecessary side effects and enhances treatment specificity. The natural origin of these products may pose challenges in terms of formulation and stability during drug preparation, which synthetic drugs, with their precise preparation processes, can more easily overcome.

Finally, a comprehensive understanding of the mechanism of action of natural products and their potential applications in the treatment of diseases such as lung cancer is crucial. This requires the application of innovative technologies such as single-cell omics and chemical proteomics to unravel the intricate interactions between natural products and the immune system, providing robust support for the discovery of new therapeutic strategies and drugs. Overall, the research and application prospects of natural products in the field of lung cancer treatment are promising. Through in-depth exploration of their biological activities and elucidation of their mechanisms of action, we aim to discover the unique potential of natural products in the treatment of lung cancer and immunotherapy. However, it is essential to acknowledge and continuously strive to overcome the challenges posed by their complexity and diversity.
